# Mapping the forage nitrogen, phosphorus, and potassium contents of alpine grasslands by integrating Sentinel-2 and Tiangong-2 data

**DOI:** 10.1186/s13007-023-01024-y

**Published:** 2023-05-15

**Authors:** Xuanfan Zhang, Tiangang Liang, Jinlong Gao, Dongmei Zhang, Jie Liu, Qisheng Feng, Caixia Wu, Zhiwei Wang

**Affiliations:** 1 State Key Laboratory of Herbage Improvement and Grassland Agro-Ecosystems, College of Pastoral Agriculture Science and Technology, Lanzhou University, Lanzhou, 730020 China; 2 State Key Laboratory of Cryospheric Science, Northwest Institute of Eco-Environment and Resources, Chinese Academy of Sciences, Lanzhou, 730000 China; 3 Guizhou Institute of Prataculture, Guizhou Academy of Agricultural Sciences, Guiyang, 550006 China

**Keywords:** Alpine grasslands, Sentinel-2 MSI, Tiangong-2 MWI, Forage quality, Remote sensing

## Abstract

Nitrogen (N), phosphorus (P), and potassium (K) contents are crucial quality indicators for forage in alpine natural grasslands and are closely related to plant growth and reproduction. One of the greatest challenges for the sustainable utilization of grassland resources and the development of high-quality animal husbandry is to efficiently and accurately obtain information about the distribution and dynamic changes in N, P, and K contents in alpine grasslands. A new generation of multispectral sensors, the Sentinel-2 multispectral instrument (MSI) and Tiangong-2 moderate-resolution wide-wavelength imager (MWI), is equipped with several spectral bands suitable for specific applications, showing great potential for mapping forage nutrients at the regional scale. This study aims to achieve high-accuracy spatial mapping of the N, P, and K contents in alpine grasslands at the regional scale on the eastern Qinghai-Tibet Plateau. The Sentinel-2 MSI and Tiangong-2 MWI data, coupled with multiple feature selection algorithms and machine learning models, are applied to develop forage N, P, and K estimation models from data collected at 92 sample sites ranging from the vigorous growth stage to the senescent stage. The results show that the spectral bands of both the Sentinel-2 MSI and Tiangong-2 MWI have an excellent performance in estimating the forage N, P, and K contents (the R^2^ values are 0.68–0.76, 0.54–0.73, and 0.74–0.82 for forage N, P, and K estimations, respectively). Moreover, the model integrating the spectral bands of these two sensors explains 78%, 74%, and 84% of the variations in the forage N, P, and K contents, respectively. These results indicate that the estimation ability of forage nutrients can be further improved by integrating Tiangong-2 MWI and Sentinel-2 MSI data. In conclusion, integration of the spectral bands of multiple sensors is a promising approach to map the forage N, P, and K contents in alpine grasslands with high accuracy at the regional scale. This study offers valuable information for growth monitoring and real-time determination of forage quality in alpine grasslands.

## Introduction

The Qinghai-Tibet Plateau's grassland area accounts for approximately 44% of all grassland in China, making it not only the most extensive high-altitude pasture in the world but also an essential production base for animal husbandry [1, 2]. The health and quality of alpine grassland are directly correlated with the sustainable utilization of grassland resources. The nitrogen (N), phosphorus (P), and potassium (K) contents of forage in alpine grasslands are crucial factors limiting grassland productivity and ecosystem function. In addition, they are also important indicators to evaluate grassland quality. N is the most abundant element in plant protein, which not only participates in the formation of plant enzymes, hormones, and chlorophyll but also has a close relationship with the photosynthesis of grassland [3–5]. It is essential for enhancing the nutrition and productivity of grasslands and plays a significant role in plant-critical activities. P is primarily involved in the synthesis of vital life substances in plants, such as nucleic acids, ATP, and phospholipids [6]. K is an indispensable mineral nutrient in plant metabolism and synthesis and can enhance plant photosynthesis and stress resistance to a certain extent [7, 8]. The traditional methods for estimating forage mineral nutrients mainly depend on field sampling and spatial interpolation techniques with relevant data, which are time-consuming, labor-intensive, and costly; in addition, they have poor spatial representations and accuracy [9, 10]. Therefore, there is an urgent need and it is particularly important to explore an effective method for accurately capturing the dynamic changes and spatial–temporal distribution patterns of forage N, P, and K contents in alpine grasslands.

With the development of remote sensing technology, multispectral, hyperspectral, and chlorophyll fluorescence data are widely used in the quantitative analysis of plant physicochemical parameters. In contrast to traditional methods, these approaches provide a more effective and practical method to estimate forage nutrient content at the regional scale [11, 12]. Previous researchers used the hyperspectral absorption features of the vegetation canopy and the corresponding vegetation index, combined with a variety of statistical models (e.g., gaussian process regression, random forest (RF), and linear mixed effects model), to obtain satisfactory estimation accuracy of plant N, P and K contents [13–15]. For instance, the variables R′708.88, R′704.85, and R′697.36 in the red-edge (RE) region had outstanding contributions to the estimations of forage N, P, and K contents, and the estimation accuracy of these parameters reached 80% [16]; the spectral bands centered at 700 nm, 710 nm, 1160 nm, 1170 nm, and 1180 nm were sensitive to crude protein (CP) content of Mediterranean grasslands and obtained a relatively high estimation accuracy of CP (coefficient of determination (R^2^) ≥ 0.7) [17]. In addition, previous studies have demonstrated that the status of plant growth and the reflectivity of the RE band had a significant relationship [18]. The feature parameter from the RE position have been widely applied to assess the quality and quantity of vegetation in farmland, forest, and grassland ecosystems [11, 19]. The RE band describes the spectral characteristics between the maximum chlorophyll absorption in the red region and the reflection peak in the near-infrared (NIR) region, which is closely related to vegetation growth and environmental stress and shows potential in the assessment of forage quality [17, 20]. For instance, Raab et al. [21] studied the ability of combined radar and multispectral data to predict the CP (R^2^ = 0.72 and RMSE = 1.70%) concentration of forage in southeastern Germany, and the results indicated that the ratio of the NIR and RE regions exhibited a strong contribution to the model performance. Therefore, remote sensing technology has been shown to be an effective way to estimate forage quality in alpine grasslands. Compared with multispectral data, hyperspectral data has richer spectral bands and thus can detect weak spectral features that cannot be detected with the utilization of traditional multispectral sensors (such as MODIS) [22]. Hyperspectral data has been extensively used for modeling estimation and spatial and temporal inversion of vegetation physicochemical parameters, such as N and chlorophyll [15, 23]. The multispectral imagery of Sentinel-2 and the hyperspectral imagery captured by a hyperspectral sensor (OCI-F-Imager) were utilized by Askari et al. [24] to estimate the CP concentration of grassland in Ireland. The results indicated that hyperspectral data (residual predictive deviation (RPD) ≥ 2.5 and R^2^ ≥ 0.8) performed better than Sentinel-2 multispectral data (estimated model performance reached a moderate level, 1.4 ≤ RPD < 2.0 and R^2^ ≥ 0.6) in the estimation of forage CP. However, due to the expense of obtaining hyperspectral satellite and airborne data, their applications in the real-time monitoring of grass growth conditions and forage nutrient estimation are greatly limited, especially at a large scale.

In recent years, a new generation of multispectral sensors (i.e., Rapid Eye and WorldView-2) equipped with RE bands significantly related to plant N and chlorophyll has shown great potential and advantages in the long-term monitoring of vegetation growth [25]. Several satellite sensors with a single RE band have been utilized for local assessments of natural grassland forage quality, and some practical and innovative results have been achieved [26–28]. However, some limitations remain for mapping alpine grassland forage nutrients with high accuracy at a large scale, such as the high data acquisition cost and limited mapping scope, which restrict the high-frequency and dynamic monitoring of forage growth conditions at the regional scale. Therefore, considering cost and feasibility, Tiangong-2 moderate-resolution wide-wavelength imager (MWI) and Sentinel-2 A/B multispectral instrument (MSI) data are more suitable for collaborative monitoring of grassland nutrients at the regional scale because their data are freely available and are easy to obtain. Moreover, the band configurations of these two data types are similar. The Tiangong-2 space laboratory launched successfully on September 15, 2016, is the second space laboratory launched by China following the successful mission of the Tiangong-1 space laboratory [29]. The MWI carried by the Tiangong-2 space laboratory, as the core sensor for Earth observation, has a high spectral and temporal resolution. It has been widely used in ecological environment protection and assessment, land cover classification, lake, and ocean monitoring, and other applications [30, 31]. Tiangong-2 MWI data have a 300 km image width and 14 bands ranging from the visible region to the NIR region, with most of these bands located in the 400–990 nm range, making them ideal for mapping large-scale vegetation growth. Two satellites, Sentinel-2 A/B, were launched in June 2015 and March 2017, respectively, and carry a MSI that covers the visible, NIR, and shortwave infrared (SWIR) regions.

Sentinel-2 MSI has three RE bands and two NIR bands, which have been shown to be closely related to plant nutrient contents, such as chlorophyll, N, and cellulose. These spectral bands have shown significant importance in estimating plant physicochemical parameters and have been extensively used in vegetation growth monitoring [32, 33]. Fernández-Habas et al. [34] utilized Sentinel-2 MSI multispectral data to retrieve the CP and neutral detergent fiber (NDF) of Mediterranean permanent grasslands and found that the variables located in the RE and SWIR were the most sensitive to CP and NDF content, and the blue and red spectral bands played a certain role in the estimation process. Using simulated Sentinel-2 data, Ramoelo et al. [35] demonstrated that the RE and SWIR regions were reliable predictive factors for spatially mapping N and CP in a savanna in southern Africa. In addition, the calculation of the N and chlorophyll contents in grassland and crops using Sentinel-2 MSI data has been explored. The MERIS terrestrial chlorophyll index, the green chlorophyll index and the RE chlorophyll index worked well in the assessment of the chlorophyll and N contents [25], and the spectral bands of MSI, which are centered on 705 nm and 740 nm were well positioned for deducing these vegetation indices. These studies attest to the value of RE bands from MSI in vegetation monitoring. Notably, the Tiangong-2 MWI has rich spectral information and a large image width, and its application in estimating the physicochemical parameters of grassland is not yet mature. Therefore, the potential of Tiangong-2 MWI data for monitoring vegetation growth at the regional scale needs to be further explored.

Traditional multivariate regression analysis methods can readily result in overfitting and are susceptible to the multicollinearity of variables [36, 37]. In addition, these methods require that the data follow a normal distribution. In contrast, machine learning algorithms, such as RF and support vector machine (SVM), can address these problems well [38]. SVM algorithm can effectively prevent the influence of high-dimensional data, small samples, and local optimization problems [23]. In the case of a small sample size, SVM is superior to the other modeling approache. The multicollinearity of variables can be reduced by the RF algorithm, which is suitable for exploring the relationships between specific vegetation biochemical parameters and multiple spectral variables [26, 39].

In addition, to our knowledge, there are very few studies that focus on using the Tiangong-2 MWI data to estimate the quality of alpine grasslands. Moreover, the feasibility of integrating Sentinel-2 MSI and Tiangong-2 MWI data to map forage nutrients in alpine grasslands has not been successfully confirmed. Our study was carried out in an alpine grassland on the eastern Qinghai-Tibet Plateau. The forage N, P, and K contents of the alpine grassland were estimated using remote sensing data combined with observation data. To do so, two feature selection algorithms (i.e., RFFS and LASSO) and two machine learning models (i.e., RF and SVM) were employed in this study. The primary goals of this paper are (1) to test whether the spectral band configuration of Sentinel-2 MSI can effectively estimate the forage N, P, and K contents in alpine grasslands; (2) to verify whether Tiangong-2 MWI has the potential to estimate the forage N, P and K contents; and (3) to explore whether the estimation performance of the forage N, P and K contents in alpine grasslands can be improved by the integrated use of Sentinel-2 MSI and Tiangong-2 MWI data.

## Materials and methods

### Study area

**Fig. 1:**
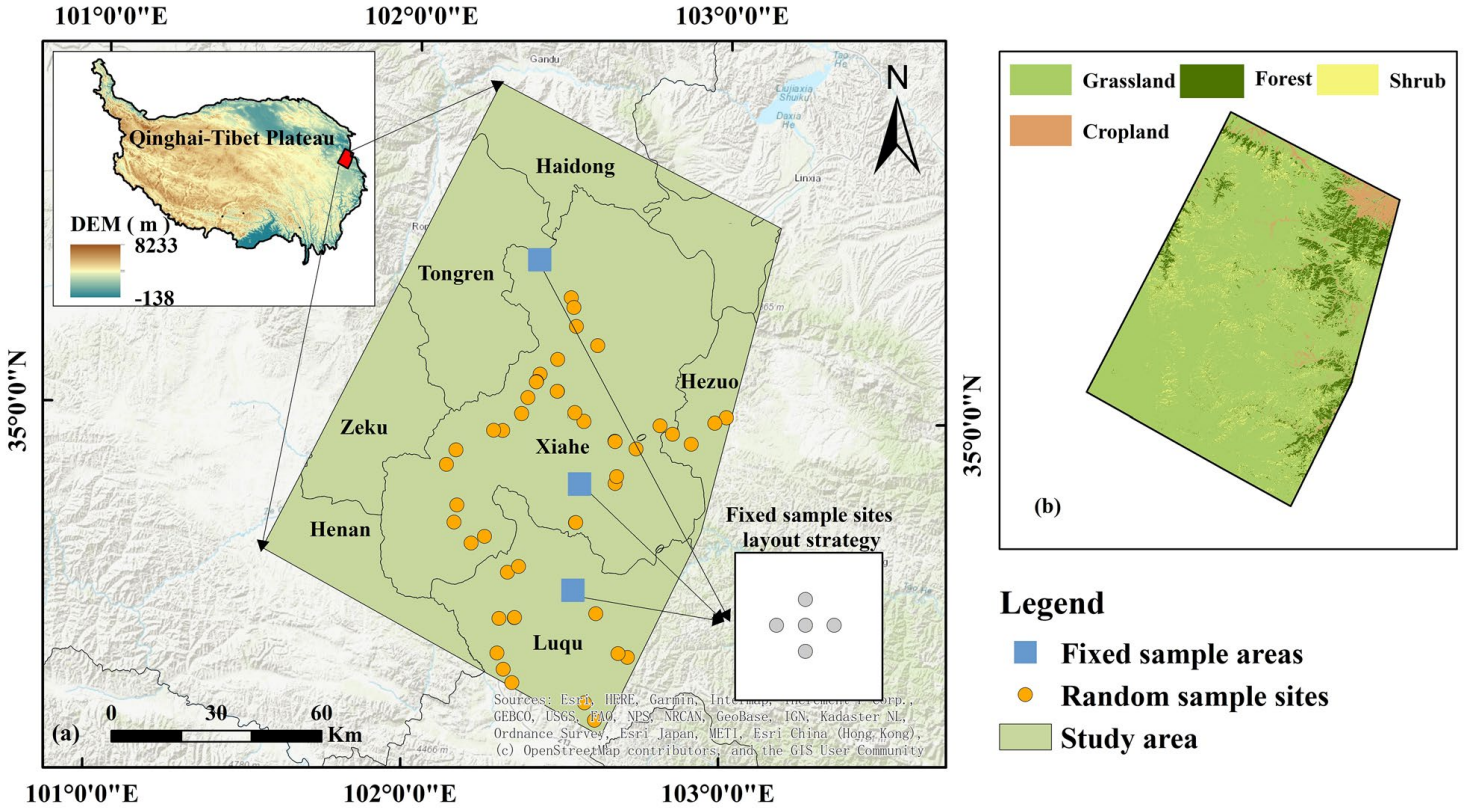
Spatial distribution of fixed monitoring sample areas and random sample sites in the study area (**a**) and land use (**b**) [41]

The study area is situated on the eastern Qinghai-Tibet Plateau with an altitude of 1966–4639 m (Fig. 1). The grassland resources in this region are rich, and animal husbandry is the leading industry. In addition, the main vegetation types in this region are grassland, forest, and shrub, among which the grassland area is the largest, providing a material guarantee for the development of animal husbandry. The primary grassland classifications in the research region include alpine meadow, mountain meadow, temperate grassland, and alpine grassland, among others. The alpine meadow dominates the surrounding area, making up 52.80% of the total grassland area in this region, and the dominant genera in this region are *Carex parvula*, *Bistorta vivipara,* and *Carex capillifolia.* The mountain meadow is the second most extensive grassland type, making up 19.32% of the grassland area in the research region overall; the predominant genera in mountain meadow are *Carex duriuscula* and *Elymus nutans.* In general, grassland vegetation mainly undergoes vegetative and reproductive growth from May to August and gradually enters the senescent stage beginning in September. The study area has a typical plateau continental climate and is located in a humid alpine climate zone. The annual average temperature ranges from 1 to 3 °C, and precipitation is abundant, with an average of between 400 and 700 mm of precipitation falling each year. The rainfall in this region is usually concentrated in July–September, and then the precipitation and temperature gradually decrease. Due to the influence of monsoons, the distribution of precipitation shows a pattern of being higher in the south and lower in the north [40]. In addition, as a result of the high average altitude, low surface pressure, complex terrain, and changeable climate, disastrous weather, including droughts and floods, cold weather in late spring, snow disasters, and cold waves occur frequently. Under the influences of overgrazing and extreme climates, combined with the extremely extensive management mode of animal husbandry, the vegetation community structure in this region has become singular, the conflict between forage and livestock has intensified, the quality of grassland has decreased, and the function of the grassland ecosystem has been seriously damaged, hindering the healthy development of animal husbandry in grassland areas.

### Grassland observation data

Three field campaigns were carried out in the study area in July (when the forage is in the vigorous growth stage), September (when the forage is gradually entering the senescent stage), and November (when the forage is completely in the senescent stage) 2017, and a total of 92 sample sites were observed (Fig. 1). According to the distribution characteristics and utilization pattern of the grassland, three typical areas were selected as fixed monitoring sample areas; these areas were located in Ganjia Township and Yaliji Township in Xiahe County and Xicang Township in Luqu County. In addition, 5 fixed sample sites (100 × 100 m) were established within each fixed sample area. Moreover, to understand the overall forage growth, some sampling sites (100 × 100 m) were randomly set up in the study area. Each sample site consisted of 5 subplots (0.5 × 0.5 m) to represent site variability. For each quadrat, the factional coverage, height, and species number of the vegetation in the community, the proportion of nonphotosynthetic vegetation, longitude, latitude, and other relevant information were first obtained. Then, the forage samples in the quadrats were cut to ground level, placed in a quadrat bag, and transported to the laboratory.

Forage samples collected from the field were dried in an oven at 65 °C for 48 h to a consistent weight; subsequently, the forage samples from the same site were mixed, crushed, and sieved, and then, their nutrient contents were quantitatively analyzed by chemical analysis. First, digestion, dehydration, carbonization, and a series of oxidation reactions were conducted on grass samples using H_2_SO_4_–H_2_O_2_ solution. The N and P contents of the forage were measured using the flow injection analyzer (Fia-star 5000, Foss Tecator, Sweden); at the same time, a flame spectrophotometer was used for quantitative analysis of the K content. The N, P, and K contents were measured using the Bertfelot reaction, phosphomolybdate blue reaction, and flame reaction, respectively [42, 43]. The chemical analysis method adopted in this study is simple to perform and has high accuracy and monitoring efficiency, making it ideal for the analysis of numerous plant samples.

### Satellite image and processing

The satellite data utilized in this study include Tiangong-2 MWI and Sentinel-2 MSI images. The Tiangong-2 MWI data were acquired from the China Manned Space Application Data Promotion Service Platform (http://www.msadc.cn/sjfw/), and the Copernicus Open Access Hub (https://scihub.copernicus.eu/) was used to gather the Sentinel-2 MSI data. The images taken closest to the time of sampling were preferentially selected in this study, and these data were nearly cloud- and cloud-shadow-free. A total of 20 Tiangong-2 MWI images and 36 Sentinel-2 MSI images were selected.

The wide-wavelength imager carried by the Tiangong-2 satellite consists of four different types of cameras (i.e., a visible/NIR camera, SWIR camera, thermal infrared camera, and visible polarized camera). The data from the visible/NIR camera were used in this study; these data consists of 14 bands with a spatial resolution of 100 m and are primarily centered between 400 and 990 nm (Table 1). The Tiangong-2 MWI images downloaded from the data platform are radiance products, that have been preprocessed with systematic radiometric correction and geometric correction. The FLAASH atmospheric correction module in ENVI 5.3 (Exiles Visual Information Solutions, USA) was utilized for image atmospheric correction by setting the orbit parameters of Tiangong-2 and importing its spectral response function. Finally, masking and resampling (20 m) were conducted for these images in ENVI 5.3. All 14 spectral bands were selected as independent variables for modeling.

**Table 1:** Spectral bands of Sentinel-2 MSI and Tiangong-2 MWI

Sentinel-2 MSI			Tiangong-2 MWI		
Band	Central wavelength (nm)	Band width (nm)	Band	Central wavelength (nm)	Band width (nm)
B2	490	65	V1	413	20
B3	560	35	V2	443	20
B4	665	30	V3	490	20
B5	705	15	V4	520	20
B6	740	15	V5	565	20
B7	783	20	V6	620	20
B8	842	115	V7	665	10
B8a	865	20	V8	682.5	10
B11	1610	30	V9	750	20
B12	2190	142	V10	820	20
			V11	865	40
			V12	905	20
			V13	940	20
			V14	980	20

The Sentinel series satellites are the component constellation launched by the ESA for the Copernicus program. The MSI multispectral sensor has 13 spectral bands with spatial resolutions of 10 m (B2, B3, B4, and B8), 20 m (B5, B6, B7, B8a, B11, and B12), and 60 m. These bands are located from 430 to 2190 nm, and the RE bands include B5, B6, and B7 (Table 1). The Sentinel-2 images downloaded from the data platform are the Level-1C orthorectified TOA reflectance product. Some initial data processing, such as atmospheric correction, geometric correction, and resampling (20 m), was conducted in SNAP (European Space Agency, France). Finally, 10 bands excluding the B1, B9, and B10 bands were selected as independent variables for modeling [44].

### Statistical methods

#### Feature band selection

Two feature band selection algorithms were adopted in this study: the first is the random forest feature selection algorithm (RFFS) [39], and the second is the least absolute shrinkage and selection operator (LASSO) algorithm proposed by statistics professor Tibshirani [45] at Stanford University in 1996. For RFFS, the variables are first ranked by their importance score, and then, the sequential backward selection method is used to eliminate the variables with the lowest score. The importance score of variables generated by each iteration can be used as the basis for feature elimination, and the R^2^ is calculated after each iteration to evaluate the model performance. The variable set with a higher R^2^ value and a smaller number of variables is selected as the final result of the feature selection after several iterations.

The LASSO is a regression method with feature selection and regularization performed simultaneously to enhance the interpretability and accuracy of statistical models [46]. In addition, the algorithm achieves compression by generating a penalty function, which is the variable coefficient. The regression coefficient of independent variables with little or no influence is compressed to 0 to solve the overfitting problem of models and effectively reduce the complexity and instability of the models.

#### Modeling

To effectively overcome the uncertainties brought about by using single regression models for prediction, two machine learning models, RF and SVM, were utilized in this study. The RF regression model is a supervised learning algorithm proposed by Breiman [47], which trains samples based on multiple decision trees and then utilizes the trained model to predict the categories of the tested samples. The bootstrap sampling method is used by the RF model to extract multiple sample sets with the same number of samples from the original samples to form sample subsets, and the number of samples in each sample subset is the same as that in the initial data sample set. Then, each sample subset is used to build a decision tree, and the predicted results of multiple decision trees are integrated to acquire the final result; thus, the final result of the established model is jointly determined by each decision tree. The two main parameters (ntree and mtry) selected the default values [48, 49].

SVM is a machine learning model developed from statistical theory that has a good function for small sample classification and regression [50]. Its goal is to establish the best classification hyperplane to improve the generalization ability of the model. Concurrently, the original data space is mapped to a higher dimensional space through the kernel function to effectively solve nonlinear problem. Due to the excellent performance of the radial basic function (RBF) in several studies, RBF was selected as the kernel function of our model [51], and the genetic algorithm was used to optimize the two key parameters in SVM. The above training and optimization of the SVM and RF models were performed using MATLAB 2016a software (Math Works, USA).

#### Accuracy assessment

The performance of the established models for forage nutrient content estimation was assessed by a tenfold cross validation (CV) procedure. All the variables were separated into ten subsets, of which nine were chosen as training data to establish the models, and one was utilized as test data (the sample size for the first nine folds, and the last fold is 9 and 11, respectively). This procedure was repeated 10 times to obtain the R^2^ and root mean square error (RMSE) between the estimated and measured values. The R^2^, normalized root mean square error (NRMSE), RMSE, and RPD were employed to assess the performance of the N, P, and K content estimation models. Each model was repeated 50 times to reduce accidental error, and the final outcome was determined by averaging all the results. Furthermore, the Akaike information criterion (AIC) and Bayesian information criterion (BIC) values were utilized for the assessment of the complexity of the models established in this study. AIC and BIC are criteria used to weigh the complexity of the established models and the goodness-of-fit of the data [52, 53]. The best model must strike the correct balance between model complexity and data fitting prowess, and this balance is determined by using both criteria. If several models have the same accuracy, the model with a smaller BIC is preferred. R^2^, RMSE, NRMSE, RPD, AIC, and BIC are calculated as follows:

1 $$ \begin{array}{*{20}c} {{\text{R}}^{2} = \left( {1 - \frac{{\mathop \sum \nolimits_{i = 1}^{n} \left( {{\text{y}}i - \hat{y}i} \right)^{2} }}{{\mathop \sum \nolimits_{i = 1}^{n} \left( {{\text{y}}i - \overline{y}} \right)^{2} }}} \right)} \\ \end{array} $$

2 $$ \begin{array}{*{20}c} {RMSE = \sqrt {\frac{{\mathop \sum \nolimits_{i = 1}^{n} \left( {yi - \hat{y}i} \right)^{2} }}{n}} } \\ \end{array} $$

3 $$ \begin{array}{*{20}c} {MSE = \frac{RMSE}{{\overline{y}}}} \\ \end{array} $$

4 $$\begin{array}{c}RPD=SD/RMSE\end{array}$$

5 $$ \begin{array}{*{20}c} {RSS = \mathop \sum \limits_{i = 1}^{n} \left( {yi - \hat{y}i} \right)^{2} } \\ \end{array} $$

6 $$\begin{array}{c}AIC=2k+n\mathrm{ln}\left(RSS/n\right)\end{array}$$

7 $$\begin{array}{c}BIC=n\mathrm{ln}\left(RSS/n\right)+k\mathrm{ln}\left(n\right)\end{array}$$

where *y*_*i*_ and ŷ_*i*_ denote the actual measured and model simulated values of forage nutrient content in the test set, respectively; ȳ denotes the average value of the actual measured forage nutrient content; $n$ denotes the sample size of the test dataset; SD is the standard deviation; RSS denotes the sum of squared residuals; and k denotes the number of model variables. According to the NRMSE grading criteria, the model accuracy is excellent when NRMSE is less than or equal to 10%, good when it is greater than 10% and less than or equal to 20%, average when it is greater than 20%, and less than or equal to 30%, and poor when it is greater than 30%. The RPD grading criteria are as follows: “better” accuracy (RPD ≥ 2.0, R^2^ ≥ 0.70), “moderate” accuracy (1.4 ≤ RPD < 2.0, R^2^ ≥ 0.60), and “poor” accuracy (RPD < 1.4, R^2^ < 0.6).

## Results

### Statistics of the forage N, P, and K contents during various growth stages

**Fig. 2:**
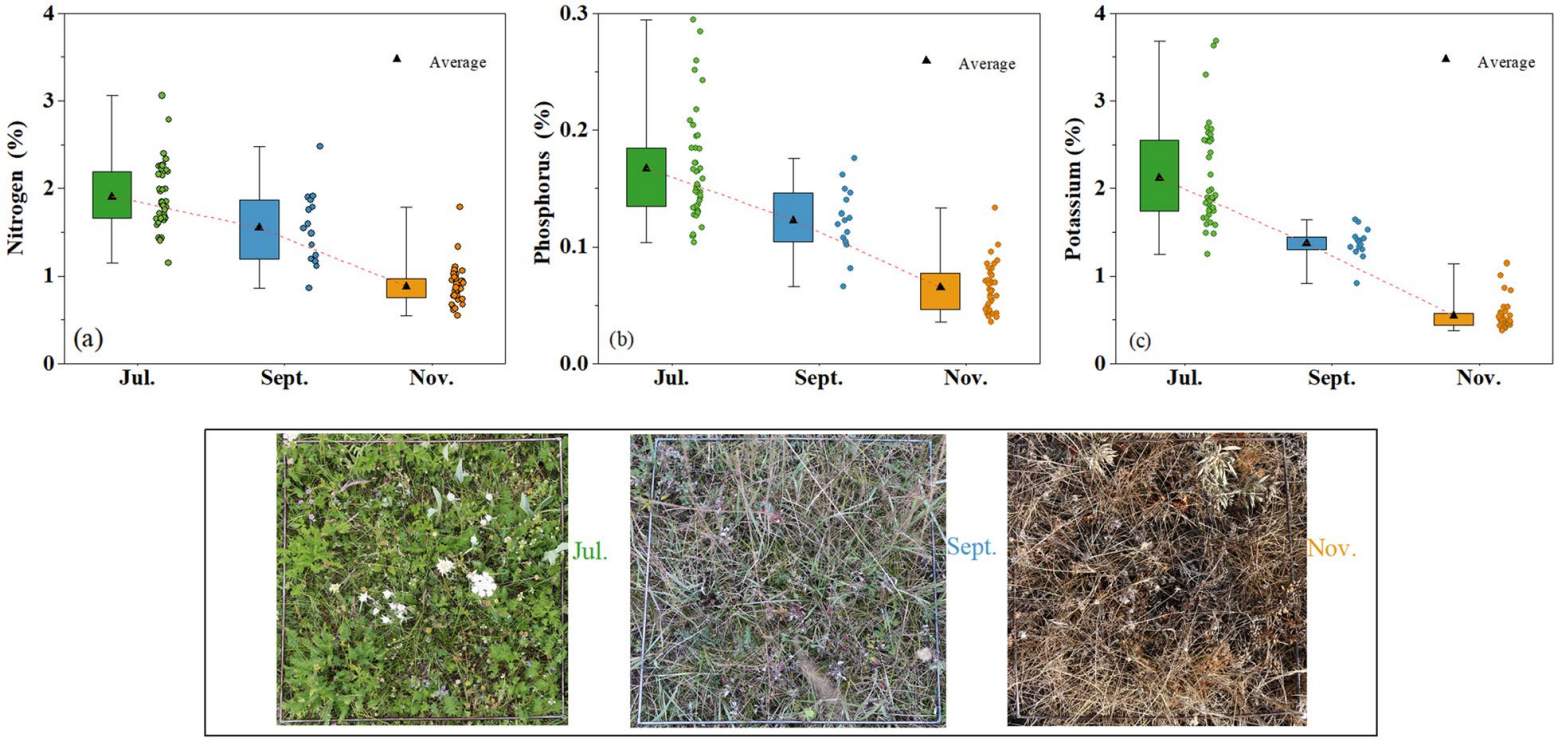
Descriptive statistics of forage N (**a**), P (**b**), and K (**c**) contents and actual photos of the alpine grassland taken in July, September, and November. The triangles indicate the mean values of the N, P, and K contents in different growth stages, and the red dashed lines are the trend lines of the mean nutrient contents

The descriptive statistics of the forage N, P, and K contents during various developmental stages and actual photos of the alpine grassland are provided in Fig. 2. As the different growth stages progress, the contents of N, P, and K decrease significantly. The average contents of N, P, and K are highest during the vigorous growth stage (1.91%, 0.17%, and 2.12%, respectively) and lowest during the senescent stage (0.88%, 0.06%, and 0.54%, respectively).

### Estimation of the forage N, P, and K contents based on Tiangong-2 MWI data

The estimation results of the N, P, and K contents in the forage obtained by utilizing various band combinations of MWI data are shown in Table 2. Compared with the model established using all 14 MWI spectral bands, the model developed by the feature bands selected by the RFFS and LASSO algorithms has higher accuracy and lower complexity (the model has low AIC and BIC). Overall, in contrast to the SVM model, the RF model yields a better estimation accuracy, demonstrating that the RF model performs better in the estimation of forage N, P and K contents. Furthermore, through comparison analysis, we find that the estimation model for the N content (NRMSE ≤ 0.20) has higher accuracy than that for the P and K contents. Among all models, the RF model with the TG-LASSO variable set as an independent variable is the optimal model for N estimation. The optimal model for P and K estimation is the RF model with the TG-RFFS variable set as the independent variable.

**Table 2:** All the forage nutrient estimation models based on the spectral bands of the Tiangong-2 MWI

TG-2	N						P						K					
	RF			SVM			RF			SVM			RF			SVM		
	TG	TG-RFFS	TG-LASSO	TG	TG-RFFS	TG-LASSO	TG	TG-RFFS	TG-LASSO	TG	TG-RFFS	TG-LASSO	TG	TG-RFFS	TG-LASSO	TG	TG-RFFS	TG-LASSO
Number	14	6	5	14	6	5	14	4	3	14	4	3	14	8	2	14	8	2
R^2^	0.76	0.73	0.75	0.67	0.68	0.68	0.73	0.70	0.69	0.61	0.60	0.54	0.81	0.81	0.79	0.77	0.74	0.74
**RMSE (%)**	0.30	0.30	0.30	0.34	0.33	0.33	0.03	0.03	0.03	0.04	0.04	0.04	0.38	0.39	0.39	0.42	0.44	0.45
NRMSE	0.20	0.21	0.20	0.23	0.23	0.23	0.27	0.28	0.28	0.32	0.36	0.33	0.27	0.28	0.28	0.30	0.32	0.32
RPD	1.56	1.59	1.65	1.62	1.65	1.66	1.48	1.54	1.49	1.44	1.28	1.38	1.69	1.74	1.85	1.77	1.72	1.73
AIC	− 155.68	− 175.42	− 184.12	− 162.64	− 182.23	− 184.66	− 565.1	− 592.53	− 589.5	− 560.73	− 574.73	− 558.97	− 103.92	− 121.53	− 145.33	− 112.02	− 119.1	− 132.51
BIC	− 120.38	− 160.29	− 175.51	− 127.34	− 167.1	− 172.05	− 529.8	− 582.45	− 581.94	− 525.42	− 567.17	− 548.89	− 68.61	− 101.36	− 140.29	− 76.72	− 98.93	− 127.47

The values of R^2^, RMSE, NRMSE, RPD, AIC, and BIC are the averages of 50 cycles of a tenfold CV. The R^2^ value is provided by the validation dataset. The number refers to the number of spectral bands used for modeling. TG-LASSO and TG-RFFS denote the set of suitable MWI spectral bands screened based on the LASSO and RFFS algorithms, respectively. *N* nitrogen, *P* phosphorus, *K* potassium, *TG* Tiangong-2)

**Fig. 3:**
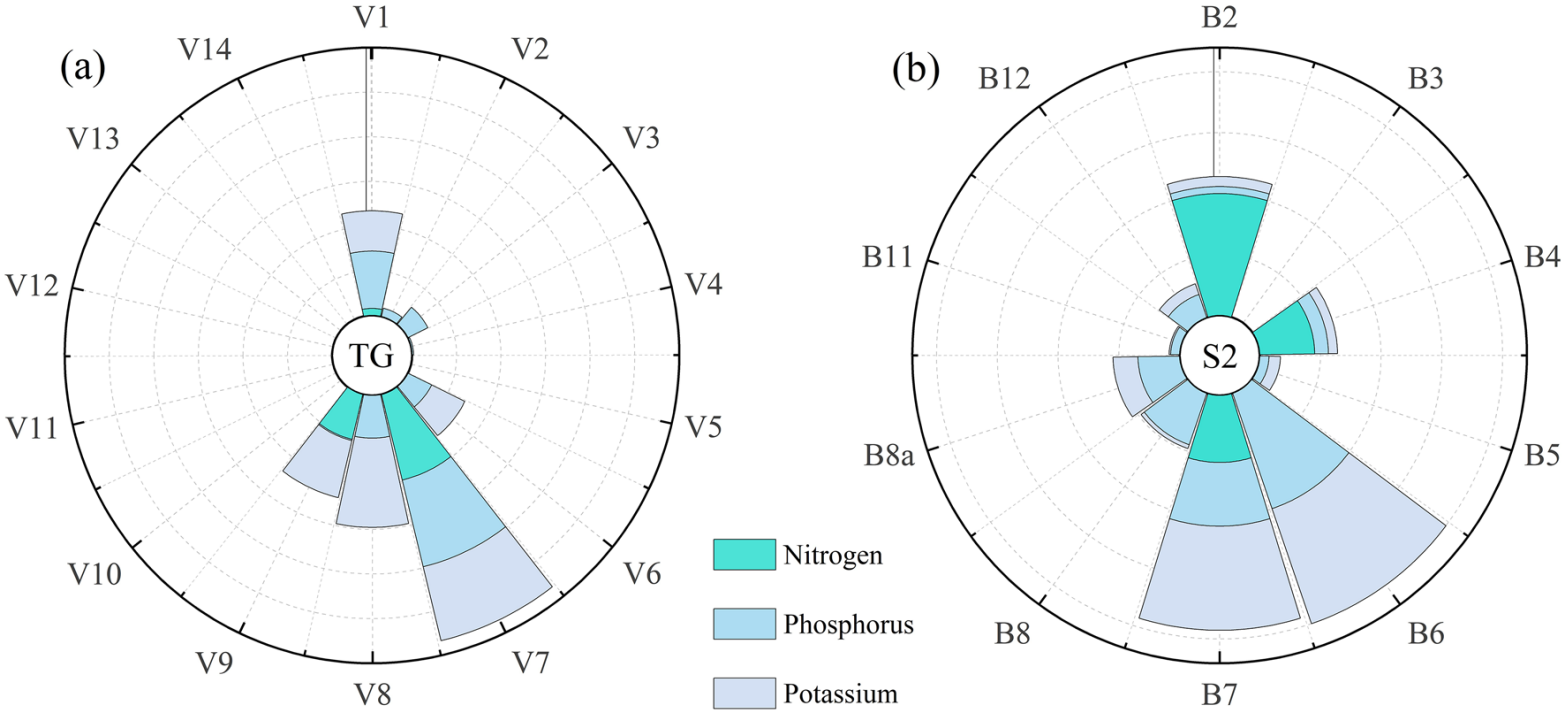
Analysis of the relative importance of different forage nutrient differences to Tiangong-2 MWI (**a**) and Sentinel-2 MSI (**b**) spectral bands. For comparison, the importance of both MSI and MWI spectral bands for nitrogen, phosphorus, and potassium was normalized to between 0 and 1. *TG* Tiangong-2, *S2* Sentinel-2

The importance score of various bands from the Tiangong-2 MWI for the estimation of the forage N, P, and K contents is illustrated in Fig. 3. The V1 (blue), V7 (red), and V8 (RE) bands are chosen as sensitive variables in the estimation of forage N, P and K contents; in particular, V7 (red) band has a higher importance in the estimation model. However, although the V1 (blue) and V8 (RE) bands are less important in the estimation models, their contributions should not be ignored. The V9 (RE) band has a prominent contribution to the estimation of the forage N and K contents but does not have a significant contribution to the estimation of the P content. Furthermore, the V6 (red) band is of some importance for estimating the P and K contents of forage, while the V3 (blue) band has a weak contribution to estimating the P content. In summary, the spectral bands covering the blue, red, and RE regions are most suitable for estimating the N, P, and K contents are most sensitively estimated.

### Estimation of the forage N, P, and K contents based on Sentinel-2 MSI data

The forage N, P, and K content estimation results utilizing different band combinations of MSI data are shown in Table 3. Compared with the model established using all 10 MWI spectral bands, the model developed by the feature bands selected by the RFFS and LASSO algorithms has a marginally higher accuracy and a higher operating efficiency (the AIC and BIC values are low). Overall, compared to the SVM model, the RF model has a higher estimation accuracy, indicating that the RF model is more robust in the estimation of forage nutrient contents. In summary, the RF model with the S2-LASSO variable set as independent variables is the optimal model for the N content estimation. The RF model with the S2-RFFS variable set as independent variables is the optimal model for the P and K estimation.

**Table 3:** All the forage nutrient estimation models based on the spectral bands of the Sentinel-2 MSI

Sentinel-2	N						P						K					
	RF			SVM			RF			SVM			RF			SVM		
	S2	S2-RFFS	S2- LASSO	S2	S2-RFFS	S2- LASSO	S2	S2-RFFS	S2- LASSO	S2	S2-RFFS	S2- LASSO	S2	S2-RFFS	S2- LASSO	S2	S2-RFFS	S2- LASSO
Number	10	7	4	10	7	4	10	7	2	10	7	2	10	8	2	10	8	2
R^2^	0.76	0.76	0.76	0.70	0.68	0.69	0.73	0.71	0.67	0.62	0.61	0.64	0.82	0.80	0.77	0.76	0.76	0.74
^RMSE (%)^	0.29	0.28	0.29	0.32	0.33	0.33	0.03	0.03	0.04	0.04	0.04	0.04	0.37	0.39	0.41	0.43	0.42	0.44
NRMSE	0.20	0.19	0.20	0.22	0.23	0.22	0.27	0.28	0.29	0.32	0.32	0.31	0.26	0.28	0.30	0.30	0.30	0.32
RPD	1.68	1.75	1.70	1.71	1.66	1.67	1.53	1.52	1.49	1.44	1.44	1.52	1.81	1.76	1.70	1.82	1.81	1.76
AIC	− 177.16	− 190.92	− 192	− 180.94	− 180.63	− 187.81	− 579.9	− 584.47	− 591.37	− 568.51	− 574.45	− 593.92	− 124.42	− 123.48	− 129.38	− 125.56	− 129.37	− 135.36
BIC	− 151.94	− 173.26	− 181.91	− 155.72	− 162.98	− 177.73	− 554.69	− 566.82	− 586.33	− 543.29	− 556.8	− 588.88	− 99.2	− 103.31	− 124.33	− 100.35	− 109.2	− 151.94

The values of R^2^, RMSE, NRMSE, RPD, AIC, and BIC are the averages of 50 iterations of a tenfold CV. The R^2^ value is provided by the validation dataset. The number refers to the number of spectral bands used for modeling. S2-LASSO and S2-RFFS denote the set of suitable MSI spectral bands screened based on the LASSO and RFFS algorithms, respectively. *N* nitrogen, *P* phosphorus, *K* potassium, *S2* Sentinel-2

The importance score of various bands from Sentinel-2 MSI in the estimation of the forage N, P, and K contents are shown in Fig. 3. B7 (RE) is selected as the suitable band for the estimation of all nutrient contents and has a strong contribution to the estimation model. The B2 (blue) and B4 (red) bands play important roles in estimating the forage N content but contribute little to estimating the P and K contents. The B11 (SWIR) band is also selected as a suitable band in the estimation of the N content; although its importance score is low, its contribution should not be ignored. In addition, the B6 (RE) band has good potential for the estimation of the forage P and K contents. In summary, the spectral bands sensitive to the estimation of the N content are located in the blue, red, RE, and SWI regions. The spectral bands with outstanding contributions to the estimation of P and K contents are mostly located in the RE region. In addition, the spectral variables from the red, NIR, and SWIR regions also play an important role in the estimation of P and K contents.

### Estimating the forage N, P, and K contents by integrating Sentinel-2 MSI and Tiangong-2 MWI data

**Fig. 4:**
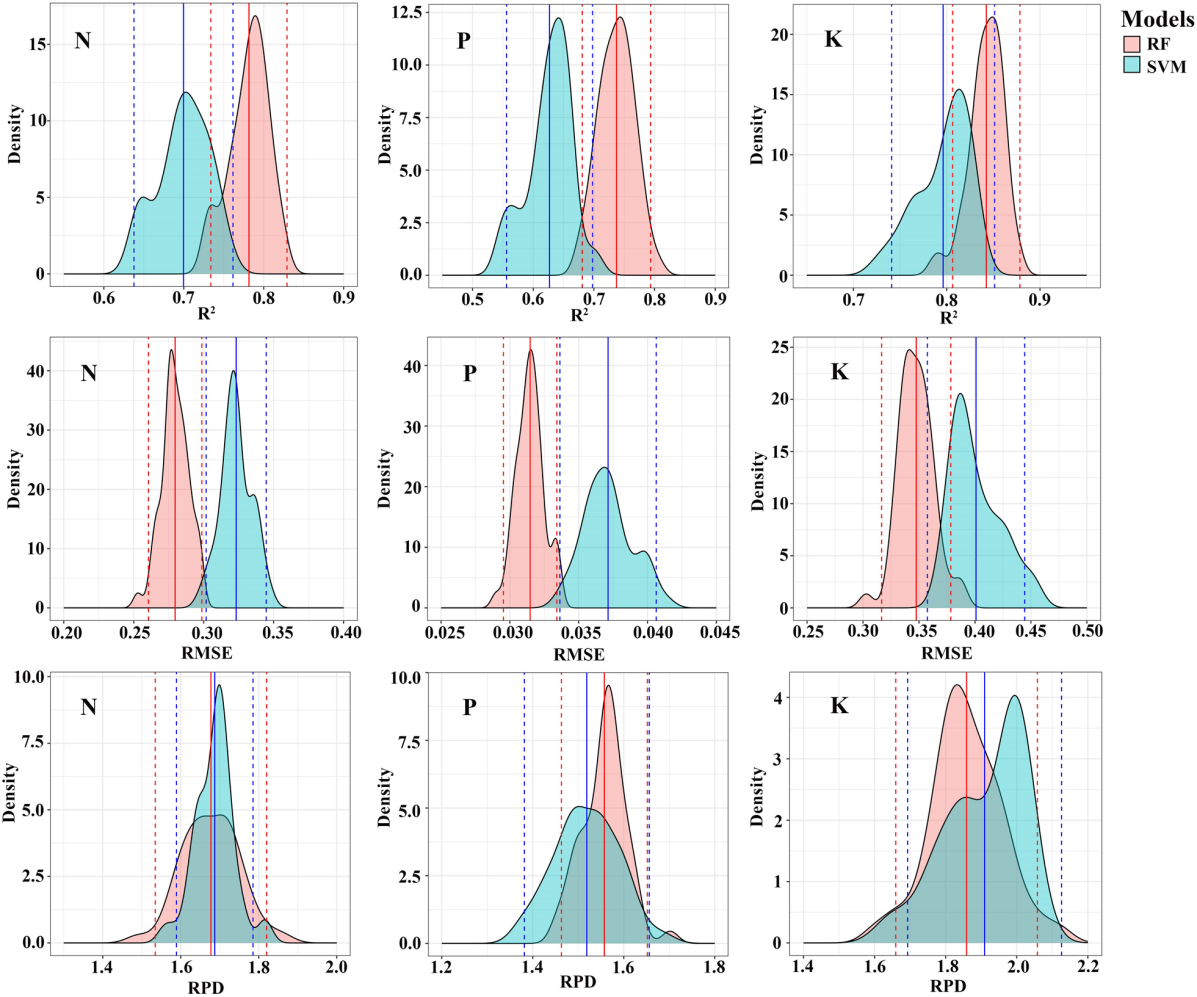
Density distribution of the R^2^, RMSE, and RPD in the validation dataset (30% of the data) according to the SVM and RF models. The solid line indicates the average and the dashed line indicates the confidence interval (2.5 and 97.5 percentile)

The spectral bands of the MSI and MWI sensors are integrated to establish the models for the estimation of the forage N, P, and K contents, and the model output results are shown in Table 4. Compared with the use of spectral bands from a single sensor (MSI or MWI), the combined use of the MSI and MWI spectral bands for estimating all nutrient contents has a higher accuracy. The estimation accuracy (R^2^) of the N, P, and K contents increases by 0.04, 0.04, and 0.03, and the NRMSE decreases by 0.01, 0.02, and 0.02, respectively; in addition, the RMSE of the N and K content estimation models decreases by 0.01 and 0.03 with the combined use of the MSI and MWI data. These results indicate that the model performance for estimating the forage N, P, and K contents can be marginally improved through the integration of multisource remote sensing data. In comparison, the RF model is more robust than the SVM model (Fig. 4 shows higher R^2^ and lower NRMSE). In the RF models established with the variable set obtained by the feature selection algorithm, the estimation models of the N and K contents perform slightly better than those of the P content. The R^2^ values of the K content estimation models are the highest, with values in the range of 0.83 to 0.85, and the R^2^ values of the N and P content estimation models are in the ranges of 0.78–0.80 and 0.72–0.77, respectively. The N content estimation models perform at a good level (0.10 < NRMSE ≤ 0 0.20), while the P and K content estimation models perform at an average level (0.20 < NRMSE ≤ 0.30). In terms of predictive ability, the models estimating all kinds of forage nutrient contents perform at a moderate level (1.4 ≤ RPD < 2.0 and R^2^ ≥ 0.60).

**Table 4:** All the forage nutrient estimation models based on the spectral bands of the MSI and MWI

S2TG	N										P										K									
	RF					SVM					RF					SVM					RF					SVM				
	TG	S2	S2TG	S2TG-RFFS	S2TG-LASSO	TG	S2	S2TG	S2TG-RFFS	S2TG-LASSO	TG	S2	S2TG	S2TG-RFFS	S2TG-LASSO	TG	S2	S2TG	S2TG-RFFS	S2TG-LASSO	TG	S2	S2TG	S2TG-RFFS	S2TG-LASSO	TG	S2	S2TG	S2TG-RFFS	S2TG-LASSO
Number	14	10	26	13	9	14	10	26	13	9	14	10	26	11	5	14	10	26	11	5	14	10	26	16	4	14	10	26	16	4
R^2^	0.76	0.76	0.8	0.78	0.78	0.67	0.7	0.7	0.7	0.7	0.73	0.73	0.77	0.74	0.72	0.61	0.62	0.64	0.62	0.63	0.81	0.82	0.85	0.84	0.83	0.77	0.76	0.77	0.79	0.8
RMSE (%)	0.3	0.29	0.28	0.28	0.28	0.34	0.32	0.32	0.32	0.32	0.03	0.03	0.03	0.03	0.03	0.04	0.04	0.04	0.04	0.04	0.38	0.37	0.34	0.35	0.36	0.42	0.43	0.42	0.4	0.4
NRMSE	0.2	0.2	0.19	0.19	0.19	0.23	0.22	0.22	0.22	0.22	0.27	0.27	0.25	0.26	0.27	0.32	0.32	0.31	0.32	0.31	0.27	0.26	0.24	0.25	0.26	0.3	0.3	0.3	0.28	0.29
RPD	1.56	1.68	1.65	1.64	1.68	1.62	1.71	1.72	1.68	1.69	1.48	1.53	1.55	1.56	1.51	1.44	1.44	1.51	1.47	1.52	1.69	1.81	1.79	1.86	1.82	1.77	1.82	1.84	1.92	1.91
AIC	− 155	− 177	− 146	− 167	− 179	− 163	− 181	− 154	− 172	− 180	− 565	− 580	− 554	− 581	− 587	− 561	− 569	− 549	− 570	− 588	− 104	− 124	− 95	− 118	− 138	− 112	− 126	− 100	− 124	− 146
BIC	− 120	− 152	− 86	− 134	− 156	− 127	− 156	− 93	− 139	− 157	− 530	− 555	− 493	− 553	− 574	− 525	− 543	− 488	− 542	− 576	− 69	− 99	− 35	− 77	− 128	− 77	− 100	− 39	− 84	− 136

The values of R^2^, RMSE, NRMSE, RPD, AIC, and BIC are the averages of 50 iterations of a tenfold CV. The R^2^ value is provided by the validation set data. The number refers to the number of spectral bands used for modeling. S2TG is the set of variables combining the MSI spectral bands and MWI spectral bands used for modeling, and S2TG-RFFS and S2TG-LASSO are the sets of suitable spectral bands from the MWI and MSI that were screened by the RFFS and LASSO algorithms, respectively. *N* nitrogen, *P* phosphorus, *K* potassium, *S2* sentinel-2, *TG* Tiangong-2

**Fig. 5:**
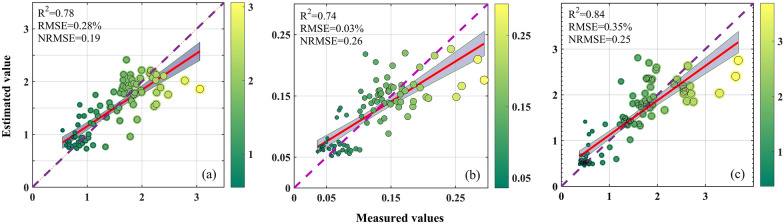
Scatter plots of measured nutrient content and nutrient content (%) estimated from optimal estimation models: optimal nitrogen estimation model based on S2TG-LASSO (**a**); optimal phosphorus (**b**) and potassium (**c**) estimation models based on S2TG-RFFS. The purple dashed line represents the 1:1 line, and the gray area represents the 95% confidence interval

**Fig. 6:**
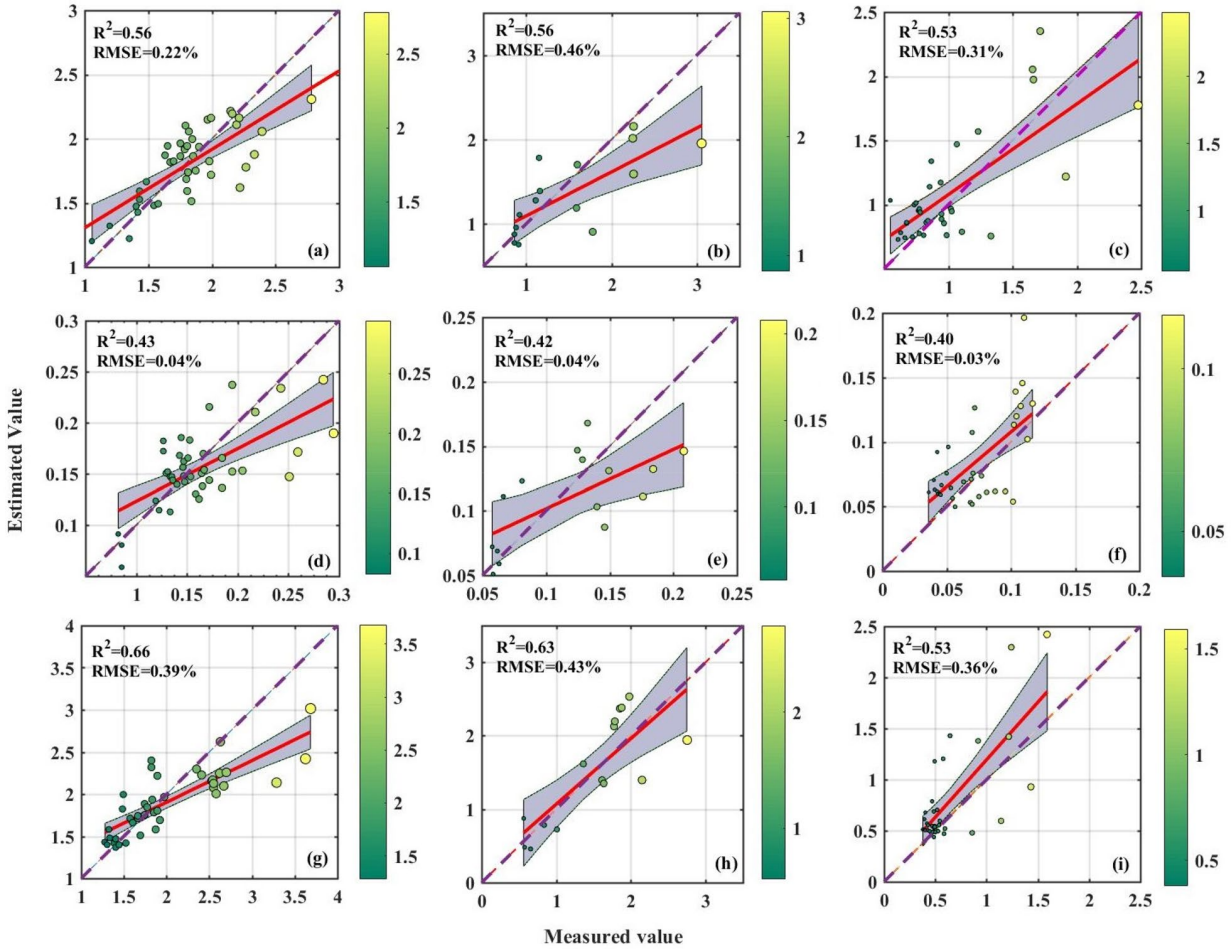
Relationships between actual observations of forage N, P and K and optimal model simulations for different grassland growth periods: (**a**) N in July, (**b**) N in September, (**c**) N in November, (**d**) P in July, (**e**) P in September, (**f**) P in November, (**g**) K in July, (**h**) K in September, and (**i**) K in November (the size and color of the circles in the figure are related to the actual observations of forage N, P, and K). The red line indicates the linear fit of the model-simulated values to the actual observations. The red line shows the results of the linear fit between model simulations and actual observations, and the gray area represents the 95% confidence interval

Considering the complexity and efficiency, the model with the lowest AIC and BIC is always selected as the optimal model when the accuracy of the models is similar. Consequently, taking the models' functionality, stability, and simplicity into account, the model occupied with the RF and LASSO algorithms is finally confirmed as the optimal model for forage N content estimation, and the model occupied with the RF and RFFS algorithm is confirmed as the optimal estimation model of the forage P and K contents. To evaluate the accuracy of the optimal estimation models, the estimated values are contrasted with the actual measured values. In Fig. 5, the points of the estimated values of the optimal model *vs*. the measured values are uniformly distributed on both sides of the 1:1 line, indicating an excellent fitting result. The optimal estimation model of the N content has the best fitting result with an NRMSE of 0.19, while the optimal estimation models of the P and K contents have a fitting result with an average level, with NRMSE values of 0.26 and 0.25, respectively. To further evaluate the prediction accuracy of the determined optimal model in different grassland vegetation growth stages, we compare the observed values with the simulated results (Fig. 6). The results showed that the optimal estimation models for forage N, P, and K presented satisfactory accuracy for different growth stages (July to November) (R^2^ are 0.53–0.56, 0.40–0.43, and 0.53–0.66, respectively). This indicates that the inversion model developed in this study is expected to be used to estimate the N, P, and K contents in different growth stages of alpine grassland.

### Spatial prediction

**Fig. 7:**
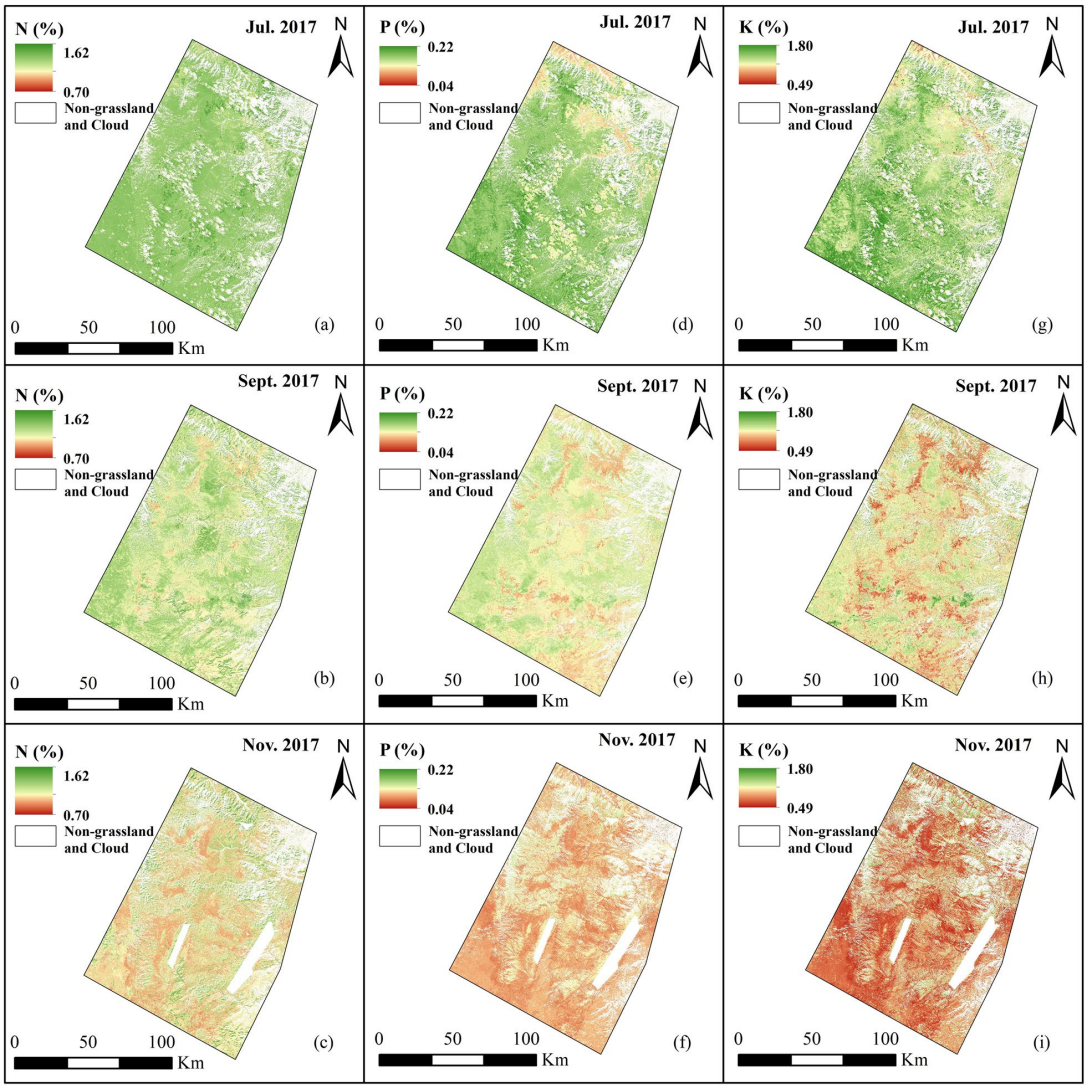
Spatial predictions of nitrogen (N) (**a–c**), phosphorus (P) (**d–f**), and potassium (K) (**g–i**) in July, September, and November 2017 using optimal estimation models (Fig. 5). The white area is where the nongrassed areas and clouds were removed

**Fig. 8:**
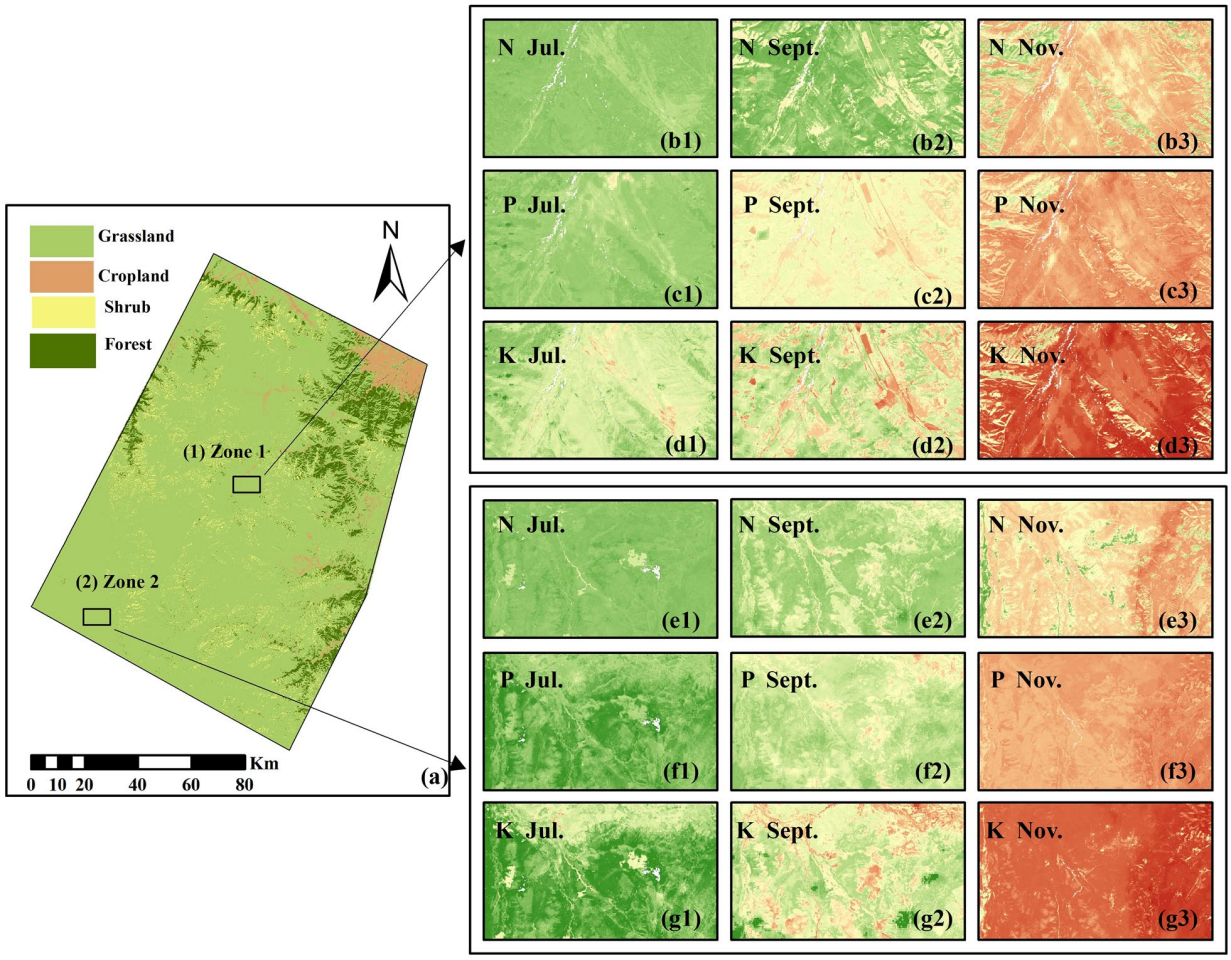
Inverse detail maps of the different growth stages of nitrogen (N) (**b, e**), phosphorus (P) (**c, f**), and potassium (K) (**d, g**). **a** Location of zone1, zone2 within the study area. **b–g** Detailed maps of N, P, and K inversions at different growth stages, 1, 2, and 3 refer to July, September, and November, respectively

Based on the optimal models of various nutrient contents, the spatial distributions of the forage N, P, and K contents at different growth stages (from the vigorous growth stage to the senescent stage) in the study area are mapped (Fig. 7). As shown in Fig. 8, the spatial mapping accuracy of all nutrients is good, with distinctive ground features during different reproductive periods, and all the nutrient contents decline significantly as the reproductive period progresses. During the vigorous growth stage, a relatively even distribution is observed in the N content, and the average N content is 1.28% (SD = 0.08%). In contrast, at the beginning of and during the senescent growth stage, the spatial distribution of the N content varies greatly, with average values of 1.15% (SD = 0.12%) and 1.04% (SD = 0.15%), respectively. The variation trend of the N content is almost the same as that of the average N content at different growth stages (Fig. 2). Except for the northernmost part of the study area, the spatial mapping of the P content resembles that of the N content during the vigorous growth stage, with an average P content of 0.15% (SD = 0.03%). During the beginning of the senescent growth stage, the spatial mapping of the P content indicates a clear distinction between areas, with an average P content of 0.11% (SD = 0.02%), and a relatively high P content is concentrated in the western and central regions of the study area. The distribution of the P content during the senescent growth stage is the most even, with an average P content of 0.08% (SD = 0.02%). The distribution of the K content during the vigorous growth stage is similar to that of the P content, and the average K content is 1.40% (SD = 0.21%). During the beginning of the senescent growth stage, the distribution of the K content is similar to that of the N content, with an average K content of 1.13% (SD = 0.22%). The distribution of the K content during the senescent growth stage is even, with an average K content of 0.89% (SD = 0.24%). In summary, the distribution of the N content during the vigorous growth stage and the distribution of the K content during the senescent stage are even. The distribution of various nutrient contents at the beginning of the senescent growth stage shows the greatest differentiation among areas, which may be due to the influence of the complex climate of the Qinghai-Tibet Plateau. In addition, natural factors, such as temperature and precipitation, in different regions differ greatly, leading to a slight difference in vegetation yellowing time.

## Discussion

### The variation of forage N, P, and K contents

The results show that the forage N, P, and K contents decreased during the vegetation growing season (July to November) (Fig. 2) [39]. This suggests that as the growth stage of grassland progresses, forage grass gradually withers, leaves tend to senesce, and the forage has a diminishing capacity to absorb mineral nutrients. Vegetative and reproductive growth occur simultaneously during the vigorous growth stage, and forage growth and development advance with vigorous metabolic activities [54]; thus, forage has a strong absorption and utilization capacity of N, P, K, and other mineral nutrients during this stage. As the forage gradually withers, plant leaves tend to senesce, vegetative growth weakens continuously, and chlorophyll decomposes gradually during the senescent stage; thus, the highly mobile N, P, and K in the leaves are gradually distributed to other organs. Moreover, the cell senescence rate exceeds the cell renewal rate; thus, the absorption capacity of forage for mineral nutrients weakens continuously. As a result, the N, P, and K contents in the aboveground part of the forage during this stage decrease to a certain extent compared with the vigorous growth stage [55]. Until the forage is completely senescent, the N, P, and K from the leaves and stems are gradually allocated to the roots, resulting in significantly lower N, P, and K contents in the aboveground part of the forage during the senescent stage compared with those in the two previous periods.

### The potential of the Tiangong-2 MWI and Sentinel-2 MSI configuration to estimate the forage N, P, and K contents in alpine grassland

**Fig. 9:**
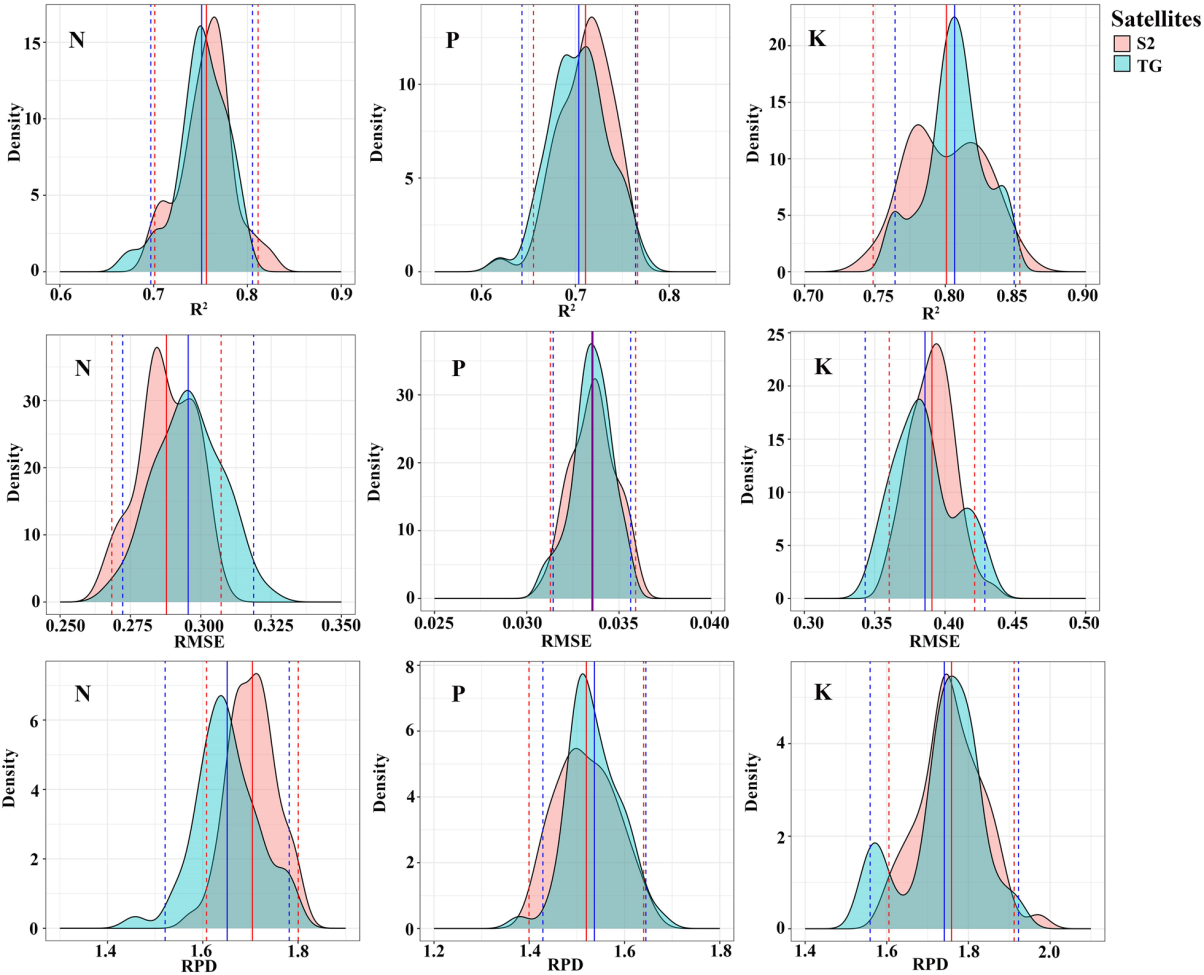
Density distribution of R^2^, RMSE, and RPD in the validation dataset (30% of the data) according to the optimal estimation model established using MSI and MWI bands. The predicted parameters are nitrogen (N), phosphorus (P), and potassium (K). The solid line indicates the mean and the dashed lines indicate the confidence intervals (2.5 and 97.5 percentiles)

In this study, Sentinel-2 MSI and Tiangong-2 MWI data are developed to estimate the N, P, and K contents of alpine grassland. In addition, the Tiangong-2 MWI and Sentinel-2 MSI data are also combined to investigate ways to further increase the accuracy of nutrient estimation in alpine grassland. These results demonstrate that compared with the use of spectral bands from a single sensor, the estimation accuracy of the N (R^2^ = 0.78), P (R^2^ = 0.74), and K (R^2^ = 0.84) contents in forage are improved by integrating the sensitive Tiangong-2 and Sentinel-2 spectral bands (Table 4). Sentinel-2 data have been widely used for vegetation monitoring, and the spectral configuration of the Sentinel-2 MSI sensor has been shown to perform well in estimating vegetation physicochemical parameters [26, 56]. In this research, the estimation models of the N, P, and K contents developed with MSI spectral bands also have ideal performances (R^2^ between 0.71 and 0.76). These results further demonstrate that the Sentinel-2 MSI data has an excellent performance in estimating the forage N, P, and K contents in alpine grasslands. As part of a new generation of multispectral sensors launched by China, Tiangong-2 MWI has abundant spectral information from the visible to the NIR region with two RE bands. The band configuration of the Tiangong-2 MWI provides a new option for monitoring forage quality in alpine grasslands. According to our results, the R^2^ values for the estimation models of various nutrient contents based on the MWI spectral bands are higher than 0.70. Moreover, the estimation accuracy of models developed using Tiangong-2 data is nearly identical to that of models based on Sentinel-2 data (Fig. 9), indicating that the Tiangong-2 sensor has the potential to estimate forage quality in alpine grasslands. In contrast to data from traditional multispectral satellites (such as Landsat and MODIS), Sentinel-2 MSI data with a high spatial–temporal-spectral resolution (10/20/60 m, 5/10 days) improves the estimation ability for vegetation physicochemical parameters [57], and the Tiangong-2 MWI data with a large image width (300 × 300 km) improve the monitoring efficiency of vegetation at a large scale.

Moreover, to meet the requirements of estimation with high accuracy for vegetation nutrients at a large scale, the integration of multisource remote sensing data is utilized to further increase the precision of nutrient estimates in alpine grasslands. The integration of multisource remote sensing data has been extensively applied to estimate the physicochemical parameters of vegetation [13, 28]. Previous studies have shown that in contrast to the estimation of acid detergent fiber and CP using derived variables alone, the performance of the model could be slightly improved by the integration of Sentinel-1 SAR and Sentinel-2 MSI data [21]. The combination of Sentinel-2 MSI spectral bands and vegetation indices increased the model performance when estimating the N:P ratio even more [23]. In addition, the N and P contents of grassland could be more accurately monitored by combining the measured canopy reflectance data and the environmental variables (e.g. climatic factors, topographic factors) [58]. The combination of multisource remote sensing data can help to highlight some significant variables, and effective information can be integrated to achieve better performance of the estimation model. The spectral band configurations of the MWI and MSI sensors are similar and complementary (Table 1), and the integration of the data from these two sensors can improve the spectral resolution of models. As a result (Table 4), the potential for the estimation of vegetation physicochemical parameters is improved. As shown in our results, by combining the MSI and MWI data, the estimated models for forage N, P, and K contents all reached a moderate level of performance (1.4 ≤ RPD < 2.0, R^2^ ≥ 0.6). The estimation accuracy of forage nutrients is marginally increased by integrating data from multiple remote sensing sources, in contrast to the single-sensor approach (the R^2^ increase by 0.04, 0.04 and 0.03 for forage N, P, and K, respectively). These results indicate that the combination of MWI and MSI data can effectively increase the predictive ability for forage nutrient content in alpine grasslands.

### The importance of MSI and MWI spectral bands in the estimation of the forage N, P, and K contents in alpine grasslands

The results of this study show that the Sentinel-2 MSI spectral bands located in the RE region (B7) and the Tiangong-2 MWI spectral band located in the red region (V7) have significant contributions to forage nutrient estimation in alpine grasslands. The spectral bands in the RE, visible, and SWIR regions have certain contributions to the N content estimation, and the spectral bands in the RE (V8 and V9), NIR (V1, B2, and B4), and SWIR (B11) regions contribute to the P and K content estimation (Fig. 3). Previous studies showed that N content was highly correlated with the spectral reflectance in the visible, RE, NIR and SWIR regions [34, 59–61], and the spectral bands sensitive to the P and K contents were mainly the RE, NIR and SWIR bands [12, 16, 62], which is consistent with the findings of our study.

The estimation of forage N content by remote sensing technology is usually based on its relationship with chlorophyll. The relationship between CP and N can also be employed for the N content estimation. In addition, the reactions of C–O, N–H, C=O, O–H, and C–H are linked to the N content within the SWIR region [24, 59, 63–65], which can explain why the Tiangong-2 MWI V1 band was selected as the feature band (absorption feature at 410 nm) [66]. Moreover, the V1 band has an important contribution to the estimation of the P and K contents. Previous research found that the bands that are more sensitive to various physicochemical parameters are often not related to their best-known absorption features but rather have some correlation with the absorption features of other parameters [12]. As P and K do not possess strong absorption characteristics in the spectrum, the screened P- and K-sensitive bands also do not always correlate with the best-known absorption characteristics but have some similarity to those of N. This finding might be a result of plants' ability to absorb N, P, and K in ways that reinforce one another. Figure 3 shows that the contribution of the B11 band to N content estimation is relatively small; this could be a result of the overall water content of leaves affecting the spectral reflectance characteristics of vegetation in the SWIR region, which obscures the influences of other biochemical substances, thus affecting the estimation accuracy and reducing the importance of this band in N content estimation [37]. Fernández-Habas et al. [34] found that Sentinel-2 B2 and B4 bands were equally important in the estimation of forage quality in Mediterranean permanent grasslands, and this finding was somewhat consistent with the results of our study. The B2 and B4 bands of the Sentinel-2 data are also selected as feature bands for the estimation of the N content in our study. In addition, the V3 band of the Tiangong-2 data is similar to the B2 band of the Sentinel-2 data, thus also playing a significant role in estimating the P content. Using hyperspectral data, Mutanga [11] found that the bands strongly related to the P and K contents of natural pasture were mainly distributed in the RE region. Moreover, the RE region is also closely related to the physicochemical parameters of vegetation. Gao et al. [16] found a significant relationship between the K content and variables derived from the RE and NIR regions, which strongly supports our findings. Moreover, similar to our results, previous studies have demonstrated that the red, NIR, and SWIR bands play significant roles in P content estimation [23, 37].

### Limitations and future prospects

This study shows that it is feasible to estimate the forage nutrient contents in the alpine grassland of the Qinghai-Tibet Plateau by using Sentinel-2 and Tiangong-2 images (R^2^ of 0.78, 0.74, and 0.84 for forage N, P, and K estimation, respectively) (Table 4). This suggests that by using multispectral satellites, the results can be expanded to regional scale mapping. By using Sentinel-2 MSI and Tiangong-2 MWI data, spatial maps of forage nutrient contents in alpine grasslands can be obtained quickly and accurately. This approach can significantly reduce the cost of forage quality surveys while providing reliable data support for herders and managers to evaluate pasture capacity, plan grazing systems in pasture areas, and fine-tune the management of grassland resources. The estimation model for the N, P, and K contents of forage in this study is influenced by grassland heterogeneity to some extent, and its performance and generalization need to be further verified in other regions. In addition, optical satellite images are susceptible to light and climatic conditions, leading to a decrease in the availability of satellite images. The dense cloudiness and frequent rainfall on the eastern edge of the Qinghai-Tibet Plateau have severely limited the study of vegetation physicochemical parameters in this region. Therefore, free, open and easily accessible multispectral remote sensing data with a high temporal resolution (e.g. Sentinel-2 and Tiangong-2) are preferred for use in this type of study, and the short revisit period of these sensors further improves the data availability in cloudy areas. The band configurations of the Tiangong-2 MWI and Sentinel-2 MSI sensors are similar, and the integration of these two sensors' data can improve the spectral resolution in a model; as a result, it is easier to capture the weak spectral information of the forage N, P, and K in alpine grasslands.

The primary goal of our investigation is to evaluate the potential of Sentinel-2 MSI and Tiangong-2 MWI configurations and integrating multisource remote sensing data to estimate N, P, and K contents. However, the performances of vegetation indices based on different sensor spectral bands in estimating forage nutrients are not discussed in this study, and thus, the integration of various vegetation indices will be taken into account in future studies. Furthermore, there is a certain correlation between ecological factors (i.e., temperature, precipitation, altitude, solar radiation, and physical and chemical parameters of soil) and the accumulation of vegetation nutrients [5, 67, 68]. For instance, a lower temperature and a higher clay content are beneficial to the accumulation of vegetation nutrients in grassland. In addition, geographical factors can reflect the difference in hydrothermal conditions and thus affect the nutrient quality of vegetation. Adequate illumination can ensure that vegetation accumulates more nonstructural carbohydrates, which is conducive to improving forage nutrients [23, 36, 69, 70]. Therefore, identifying the key ecological factors influencing vegetation nutrients and developing an estimation model of vegetation nutrients by integrating multisource remote sensing data and ecological data is a significant approach to further improve the monitoring accuracy of forage quality. Moreover, because of its high flexibility, low operating cost and small size, UAV remote sensing has been widely used in field surveys, and the combination of UAV data with multispectral data through techniques such as upscaling can further improve the estimation accuracy of vegetation physical and chemical parameters in large-scale monitoring applications [71, 72]. Other studies have also found that synthetic aperture radar (e.g., Sentinel-1) is widely used for pasture quality and quantity monitoring [21], and its independence from light and climatic conditions is particularly suitable for the Qinghai-Tibet Plateau, where clouds and rainfall are frequent. Therefore, the combined use of near-surface UAV remote sensing data, synthetic aperture radar data, multispectral satellite data and field data to monitor forage growth in alpine grasslands will be investigated in future studies.

## Conclusions

In this study, multispectral satellite data with a high spatial–temporal resolution are employed to evaluate the potential of new-generation sensors to accurately estimate and map forage N, P, and K contents in alpine grasslands at the regional scale. The following are the primary conclusions:

1. The spectral bands from the red and RE regions play a prominent role in the forage N, P, and K contents estimation. The spectral bands from the blue region are sensitive to the N content, the spectral bands from the NIR region play important roles in P and K content estimation, and those located in the SWIR region contribute less to N, P, and K content estimation.
2. Sentinel-2 MSI and Tiangong-2 MWI data perform well in the estimation of the forage N, P, and K contents in alpine grasslands. Comprehensively considering the evaluation indicators of the model, the forage N, P, and K content estimation model developed with suitable spectral bands from the Sentinel-2 MSI sensor performed better than that developed with suitable spectral bands from the Tiangong-2 MWI.
3. Compared with using spectral bands from a single sensor, the estimation performance of the forage N, P, and K contents is improved by the integrated use of suitable spectral bands from the Tiangong-2 and Sentinel-2 sensors.

In general, the selection of suitable spectral bands is very important for reducing redundancy in models. For high-accuracy growth monitoring of alpine grassland at a large scale, the focus of future studies should be to make full use of the advantages of multisource remote sensing information to develop more economical, simple, and generalized monitoring methods.

## Data Availability

The satellite data (i.e. Tiangong-2 and Sentinel-2 data) are available from the China Manned Space Application Data Promotion Service Platform (http://www.msadc.cn/sjfw/) and Copernicus Open Access Hub (https://scihub.copernicus.eu/). China's Land-Use/Cover Datasets is freely and publicly available from Wuhan University (http://doi.org/10.5281/zenodo.4417809).
